# Transcriptomic profiling and longitudinal study reveal the relationship of anti-MDA5 titer and type I IFN signature in MDA5+ dermatomyositis

**DOI:** 10.3389/fimmu.2023.1249844

**Published:** 2023-08-28

**Authors:** Yan Wang, Hongxia Jia, Wei Li, Hongping Liu, Meng Tu, Jing Li, Jiuling Cheng, Guojun Zhang

**Affiliations:** ^1^ Department of Respiratory and Critical Care Medicine, The First Affiliated Hospital of Zhengzhou University, Zhengzhou, China; ^2^ Department of Rheumatology and Immunology, The First Affiliated Hospital of Zhengzhou University, Zhengzhou, China

**Keywords:** dermatomyositis, interstitial lung disease, melanoma differentiation-associated gene 5, type I interferon, autoantibody

## Abstract

**Objective:**

This study aimed to investigate the relationship between anti-MDA5 titer and type I IFN signature in patients with MDA5+ DM.

**Methods:**

We explored the transcriptome profiling of PBMCs in MDA5+ DM patients with high-titer of antibody at disease onset or relapse and normal low-titer after treatment and healthy donors. Subsequently, we revealed the dynamic relationship between serum type I IFN scores and antibody titers.

**Result:**

Differentially expressed genes in MDA5+ DM patients were enriched for related pathways and biological functions linked to viruses and cytokines compared to healthy donors. Similar differences remained pooled between the high-titer and low-titer group, and type I-specific interferon response genes showed upregulation in high-titer group. Significant correlations were found between anti-MDA5 titers and type I IFN scores (r = 0.50, *P*< 0.001). Contemporaneous anti-MDA5 titers revealed to be significantly higher in the group with ultra-high type I IFN scores (*vs.* high group, *P* = 0.027; *vs.* low group, *P*< 0.001). Longitudinal assessment of type I IFN scores and anti-MDA5 titers, including pre- and post-treatment changes at initial diagnosis and dynamic changes during treatment, presented an asynchrony between the two parameters in response to treatment.

**Conclusion:**

Anti-MDA5 antibody titers correlated with type I IFN signature in patients with MDA5+ DM and they both changed dynamically but not synchronously over the course of treatment.

## Introduction

Anti-melanoma differentiation-associated gene 5 antibody positive dermatomyositis (MDA5+ DM), a subtype of idiopathic inflammatory myopathies (IIMs), is distinguished by a high risk of rapidly progressive interstitial lung disease (RP-ILD), scarcely clinical evidence of myopathy, and typical skin lesions. In the past two decades, the terminology of MDA5+ DM had been gradually transformed from clinically amyopathic dermatomyositis (CADM) and subsequently is defined by a centerpiece of serologic anti-MDA5 antibodies (1, 2). MDA5+ DM has a poor prognosis related to the presence of RP-ILD. An appalling proportion of patients fail to survive the first 6 or 12 months of disease onset (3, 4), but interestingly, the relapse rate is lower in surviving patients (4).

MDA5, as the autoantigen target of anti-MDA5 antibody (primitively known as anti-CADM-140), is one of three cytosolic retinoic acid-inducible gene I (RIG-I)-like receptors (RLRs), RIG-I, MDA5, and LGP2. Besides, MDA5, encoded by IFN-inducible gene (IFIH1 in human), is a key protein sensor to recognize dsRNA produced during viral infections. After binding to long-chain dsRNA, MDA5 interacts with the adapter MAVS, followed by a phosphorylation cascade that enables signaling leading to the production of IFNs and finally induces the expression of ISGs (5). In addition, type I interferon further induces MDA5 at the transcriptional level and positive feedback amplifies the interferon response (6). While type I interferon (IFN) signature has emerged as a potential factor linked to the pathogenesis of myositis, especially the MDA5 subtype. Current studies have revealed excessive type I IFN signals in peripheral blood, skin, and even muscle tissue of MDA5+ DM patients (albeit known with mild or no myopathy) (7–11).

Interestingly, clinical features of severe coronavirus disease 2019 (COVID-19) are strikingly similar to MDA5+ DM, including chest radiology, devastating “cytokine storm”, and even treatment approach (12–14). MDA5 has been proposed to be a major sensor for lung cell recognition of SARS-CoV-2 infection and governs the IFN induction in response to SARS-CoV-2 in lung epithelial cells (15, 16). Besides, anti-MDA5 antibodies are prevalent in COVID-19 patients and higher titer is related to severe disease and adverse outcomes (17). Likewise, cytokine levels, as well as antibody titers are dynamic in MDA5+ DM, and anti-MDA5 titer at diagnosis predict disease duration and correlate with disease relapse, prognosis and treatment response (4, 12–16). Similarities of both diseases may bring a deeper insight on the role of viruses and type I IFN in disease mechanism.

Therefore, we have speculated that it is the anti-MDA5 antibody, produced by viral infection or the release of RNA from impaired or aberrant cells, that may bind to MDA5 pattern recognition receptor to produce pathogenic effect, driving the production of type I interferon signaling consequently leading to inflammation and tissue damage. Here, we investigate the transcriptomic landscape of differences between MDA5+ patients with high-titer at the onset of disease and those recovered to normal low-titer after intensive treatment. Subsequent dynamic assessment of antibody titers with ensuing interferon response was performed to explore the fluctuating patterns. Findings on the interaction between anti-MDA5 antibody and type I IFN signature may provide new clues for the immunopathogenesis and potential therapeutic targets in MDA5+ DM.

## Materials and methods

### Patients

The retrospective study was conducted at the First Affiliated Hospital of Zhengzhou University (China) from January 2021 to December 2021. All MDA5+ DM patients were over 18 years old and fulfilled the 2017 EULAR/ACR Classification Criteria for Idiopathic Inflammatory Myopathies (18). Patients with overlapping disease, juvenile onset or coexistent malignancy were excluded from this study.

First, we collected peripheral blood mononuclear cells (PBMCs) for RNA sequencing (RNA-seq) from a total of 13 MDA5+ DM patients (including 8 with high-titer antibody at disease onset or relapse and 5 with normal low-titer after treatment) and 7 healthy donors (**Table 1**). Subsequently, a total of 49 patients (median age: 51 (range 18–71) years; 34 women and 15 men) were included for relationship between serum type I IFN score and antibody titer and dynamic serum evaluation (**Table 2**). There is no overlap between patients used for RNAseq analysis and patients used for measuring IFN scores. RP-ILD was defined as worsening of radiologic interstitial change, progressive dyspnoea and hypoxemia within 1 month of onset of respiratory symptoms regardless of treatment (2). This study was reviewed and approved by the Ethics Committee of the First Affiliated Hospital of Zhengzhou University (ID: 2021-KY-0301-003), Zhengzhou, China. Informed consent was obtained from all study participants.

**Table 1:** Clinical information of MDA5+ DM patients and heathy donors for RNA-seq.

Sample ID	Group	Sex	Age (years)	Disease Duration (month)	ANA titer/type	MAAs	Anti-MDA5 titer (U/ml)	CK (U/L)	Ferritin (ng/ml)	ESR (mm/h)	KL-6 (U/ml)	Treatment	Outcome
S1	High-titer	Female	51	6	1:100(±)/s+n	Ro52	194.80	32.0	3169.3	69.0	700.0	GC, CYC, TAC, JAKi, IVIg	Alive
S2	High-titer	Female	34	6	1:100(±)/h	–	101.02	104.0	743.0	34.0	1012.0	GC, TAC, JAKi, PFD	Alive
S3	High-titer	Male	36	2	1:100(-)	Ro52	182.70	32.0	1366.0	9.0	363.0	GC, CYC, CsA, IVIg	Alive
S4	High-titer	Female	50	12	1:320(+)/h+s	–	82.30	26.0	247.0	5.0	2627.0	GC, CYC, IVIg	Alive
S5	High-titer	Female	47	2	1:100(-)	–	119.76	73.0	NA	7.0	442.0	GC, MMF	Alive
S6	High-titer	Female	18	24	1:100(-)	–	108.04	82.0	751.0	26.0	NA	GC, CsA, MTX, IVIg	Alive
S7	High-titer	Male	37	7	1:100(-)	–	172.5	140.0	NA	16.0	1107.0	GC, TAC, JAKi, IVIg	Alive
S8	High-titer	Female	74	7	1:100(±)/s+c	Ro52	231.77	40.0	1956.0	107.0	672.0	GC, TAC, IVIg	Dead
S9	Low-titer	Male	40	7	1:100(-)	Ro52	25.60	33.0	290.6	2.0	441.0	GC, CYC, CsA	Alive
S10	Low-titer	Female	56	28	1:100(±)/s+c	Ro52	5.60	95.0	94.3	53.0	160.0	GC, CYC, TAC, IVIg	Alive
S11	Low-titer	Female	33	12	1:100(-)	Ro52	25.08	35.0	NA	5.0	505.0	GC, TAC, MMF, PFD	Alive
S12	Low-titer	Female	59	36	1:100(-)	–	2.10	35.0	204.0	20.0	260.0	GC, CYC, TAC	Alive
S13	Low-titer	Female	46	12	1:100(±)/s+h	Ro52	6.30	21.0	1089.7	41.0	562.0	GC, CYC, CsA	Alive
HC1~7	HD	4/3 ^a^	32 (26, 55) ^b^										

NA, not assessed. ^a^ Female/Male; ^b^ median (range). High-titer: MDA5+DM patients with high-titer of antibody at disease onset or relapse; Low-titer: MDA5+DM patients with normal low-titer (0-32U/ml) after treatment. S1, S2, S3, S4, S8 are patients at initial diagnosis.

ANA, anti-nuclear antibody test (h: homogenous, s: speckled, c: cytoplasmic, n: nucleolar); HD, healthy donors; MAAs, myositis associated autoantibodies; MDA5, melanoma differentiation-associated protein-5; CK, creatine kinase; ESR, erythrocyte sedimentation rate; KL-6, Krebs von den Lungen-6; ANA, antinuclear antibodies; GC, glucocorticoid; IVIg, intravenous immunoglobulins; CYC, cyclophosphamides; TAC, tacrolimus; CsA, cyclosporine A; JAKi, janus kinase inhibitors; MMF, mycophenolate mofetil; MTX, methotrexate; PFD, pirfenidone.

**Table 2:** The clinical data of anti-MDA5 patients at the time of specimen detecting.

	anti-MDA5+ (N=49)
Age, mean ± SD, years	50.76 ± 11.07
Sex (female/male)	34/15
Time from diagnosis to blood specimen, median (range), months	1 (0, 28)
Former or current smoker, n (%)	3 (6.1%)
BMI, median (IQR), kg/m^2^	23.95 (21.60, 26.45) ^a^
Myasthenia, n (%)	35 (71.4%)
Rash, n (%)	41 (83.7%)
cough or dyspnea on exertion, n (%)	39 (79.6%)
Arthritis, n (%)	29 (59.2%)
ALT, median (IQR), U/L	38.00 (25.00, 69.00)
AST, median (IQR), U/L	41.00 (25.50, 67.00)
CK, median (IQR), U/L	40.00 (26.25, 94.75) ^b^
ESR, median (IQR), mm/h	24.00 (9.00, 54.50)
CRP, median (IQR), mg/L	1.72 (1.00, 7.30)
Ferritin, median (IQR), ng/ml	528.00 (217.00, 1045.80) ^c^
KL-6, (IQR), U/ml	1012.00 (518.00, 1607.00) ^d^
PaO_2_, (IQR), mmHg	75.70 (67.78, 85.28) ^e^
ANA>1:100, n (%)	7 (14.3%)
Anti-Ro52 positive, n (%)	30 (61.2%)
I IFN score, median (IQR)	6.85 (0.91, 49.06)
Anti-MDA5 titer, median (IQR), U/ml	158.90 (102.46, 187.42)
Anti-MDA5 titer at initial diagnosis, median (IQR), U/ml	170.40 (145.40, 195.53)
FVC% predicted, median (IQR), %	82.50 (63.25, 101.15) ^f^
DLco% predicted, median (IQR), %	53.10 (43.40, 70.35) ^f^
HRCT	
OP pattern, n (%)	20 (40.8%)
NSIP pattern, n (%)	27 (55.1%)
Treatment	
GCs, n (%)	51 (100%)
Initial GCs dosage, median (range), mg/day	100.00 (50, 200)
IVIg, n (%)	43 (87.8%)
Tacrolimus, n (%)	33 (67.3%)
Cyclosporine A, n (%)	7 (14.3%)
CYC, n (%)	25 (51.0%)
JAKi, n (%)	19 (38.8%)
Antifibrotic drugs, n (%)	19 (38.8%)
Others, n (%)	5 (10.2%)
RP-ILD within one year, n (%)	15 (30.6%)

Antifibrotic drugs: included pirfenidone or nintedanib; JAKi: included tofacitinib; Others refer to methotrexate, mycophenolate mofetil, hydroxychloroquine or tocilizumab; GCs dosage: converted to prednisone dosage.

a Data available for 33 patients;

b 44 patients;

c 45 patients;

d 47 patients;

e 30 patients;

f 33 patients.

BMI, body mass index; PaO2, arterial oxygen pressure; ALT, alanine aminotransferase; AST, aspartate aminotransferase; CK, creatine kinase; ESR, erythrocyte sedimentation rate; CRP, C-reactive protein; KL-6, Krebs von den Lungen-6; ANA, antinuclear antibodies; anti-MDA5, anti-melanoma differentiation-associated protein-5; IFN, interferon; FVC, forced vital capacity; Dlco, diffusing capacity of carbon monoxide; HRCT, high-resolution computed tomography, NSIP, nonspecific interstitial pneumonia, OP, organizing pneumonia, GGO, ground-glass opacity; GCs, glucocorticoids; IVIg, intravenous immunoglobulins; CYC, cyclophosphamide; JAKi, janus kinase inhibitors.

### Clinical data and clinical specimens

Patients’ demographic information, as well as clinical manifestation, laboratory data, pulmonary function tests, high resolution CT (HRCT) and treatment were recorded and are listed in **Table 1**. Peripheral blood samples were collected from patients admitted in the Department of Rheumatology or the Department of Respiratory and Critical Care Medicine at the First Affiliated Hospital of Zhengzhou University. Serum was isolated by centrifugation and stored at −80°C. Peripheral blood mononuclear cells (PBMCs) were isolated using Ficoll-Paque PLUS (Cytiva, Uppsala, Sweden) density gradient centrifugation.

### Autoantibody testing

The anti-MDA5 antibodies were performed by enzyme linked immunosorbent assay kits (MBL, Japan) according to the manufacturer’s protocol. The positive cut-off value for anti-MDA5 titer was ≥ 32 U/ml. Anti-Ro52 antibody was detected using lining immunofluorescence (Euroimmun, Germany). Antinuclear antibodies (ANAs) were performed by indirect immunofluorescence on HEp-2 cells.

### RNA sequencing

RNA sequencing was performed on PBMCs. RNA was prepared with TRIzol (Invitrogen, USA) and quantified using NanoDrop. RNA purity was measured using NanoPhotometer spectrophotometer, and integrity was examined using Agilent 2100 bioanalyzer. Sequencing libraries were prepared using NEBNext® UltraTM RNA Library Prep Kit following manufacturer’s recommendations and index codes were added to attribute sequences to each sample. The libraries were sequenced on Illumina NovaSeq 6000 Systems and 125 bp/150 bp paired-end reads were generated.

### Differential expression genes analysis and functional enrichment analysis

Statistical analysis of DEGs was performed using the DEseq2 R package (1.16.1). |log2FoldChange| > 1 and adjusted *P*-value< 0.05 were assigned as the threshold for comparison of MDA5+ DM and healthy donors, and *P*-value< 0.05 for antibody titer subgroups. DEGs were subjected to enrichment analysis of their Gene Ontology (GO) functions and Kyoto Encyclopedia of Genes and Genomes (KEGG) pathways. A heatmap of eight type I-specific interferons response genes was performed to plot the difference in interferon expression between MDA5+/HD and High-titer/Low-titer samples. High-titer/Low-titer was defined according to positive cut-off value. The eight representative genes we extracted are as follows: LY6E, HERC5, IFI44L, ISG15, MX1, MX2, EPSTI1, and RSAD2 (19). Lastly, we used the ssGSEA to calculate the enrichment fraction matched to the pathway gene set for each sample and plotted the heatmap.

### Analysis of serum type I IFN signature

Given the poor reproducibility of the ELISA assay for serum type I interferon and the higher sensitivity of the functional assay compared to it (20), we used the reporter cell assay, known as WISH cells, to detect serum type I interferon scores. Human WISH epithelial cell line cells (CCL-25; American Type Culture Collection, Manassas, VA) were cultured 37°C and 5% CO2 in EMEM(30-2003, ATCC) supplemented with 10% FBS (CLARK Bioscience, USA) and 1% penicillin/streptomycin. The classical measurement of serum type I IFN activity and calculation of the score was performed as previously described (21, 22). In short, WISH cells were cultured in patient serum (50%) to stimulate for 6 hours. Subsequently, total cellular RNA was extracted using the RNeasy Mini Kit (Qiagen, Germany) and reverse transcribed into cDNA using the Revert Aid First Strand cDNA Synthesis Kit (Thermo Scientific, USA). The expression of three type I interferon-stimulated genes, MX1, IFIT1 and PKR, was detected by qPCR (Maxima SYBR Green qPCR Master Mix, Thermo Scientific, USA) to represent the induction of type I IFN.

The relative expression of type I IFN was first normalized by the relative mRNA expression of the same genes in unstimulated WISH cells, then normalized to healthy donor serum and summed to a total score to reflect the capacity of serum to upregulate the expression of IFN-induced genes. The calculation formula and primer sequences are detailed in **Supplementary Table S1**.

### Statistical analysis

Continuous variables were expressed as the mean ± standard deviation (SD). Median and interquartile range (P25, P75) or range (Min, Max) for distributed data. Categorical variables are presented as numbers (percentages). Comparisons were analyzed using the t-test or Mann-Whitney *U* test, when appropriate. Tukeys’ multiple comparisons test was used for multiple comparisons. Paired t-tests were used to compare changes before and after treatment. Cumulative rates were estimated by the Kaplan-Meier test. Correlation coefficients were established by Spearman’s correlation. SPSS (version 26.0, IBM) was used for statistical analysis, and GraphPad Prism (version 9.0) and Origin Pro 2021 were used for figure plotting. All statistical tests were two-tailed and *P*-value< 0.05 was considered statistically significant.

## Results

### Transcriptome analyze of PBMCs from MDA5+ and control groups

**Figure 1:**
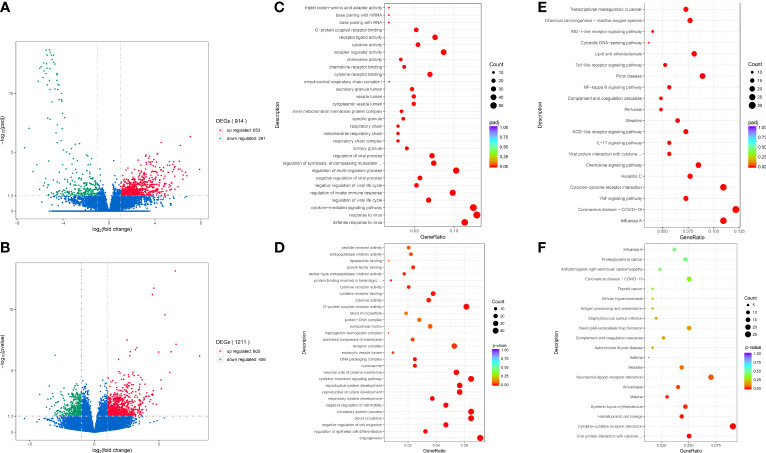
Analysis and enrichment of differentially expressed genes between different groups. Volcano plot for differentially expressed genes of MDA5+ vs. HD **(A)**; HT vs. LT **(B)**. GO enrichment scatterplot of MDA5+ vs. HD **(C)**; HT vs. LT **(D)**. KEGG enrichment scatterplot of MDA5+ vs. HD **(E)**; HT vs. LT **(F)**. MDA5+, patients with MDA5+ DM; HD, healthy donors; HT, MDA5+ patients with high-titer of anti-MDA5 antibodies; LT, MDA5+ patients with low-titer of anti-MDA5 antibodies.

We performed differential transcriptome analysis between 13 MDA5+ DM patients (including 8 with high-titer antibody at disease onset or relapse and 5 with normal low-titer after treatment) and 7 healthy donors. A total of 914 DEGs (|log2FC| > 1, adjusted *P*< 0.05) were identified that included 653 upregulated genes and 261 downregulated genes in MDA5+ DM patients compared with healthy controls (**Figure 1**). Then, in terms of anti-MDA5 antibody titer, low-titer and high-titer group displayed a similar transcriptional profile, thus we adapted the adjusted *P*-value to *P*-valued. There were 805 genes significantly upregulated (|log2FC| > 1 and *P*< 0.05), and 406 genes significantly downregulated (|log2FC| > 1 and *P*< 0.05) in the high-titer group compared with low-titer (**Figure 1**). Next, we mapped DEGs to known GO biological processes and KEGG pathways. In MDA5+ DM groups, related pathways and biological function regarding virus and cytokine were enriched for the differentially expressed mRNAs, compared with HD group (**Figure 1**). To note, similar differences remained pooled in viral and cytokine-related genes between the high-titer and low-titer group.

### Interferon gene signature in PBMCs of MDA5+ DM

**Figure 2:**
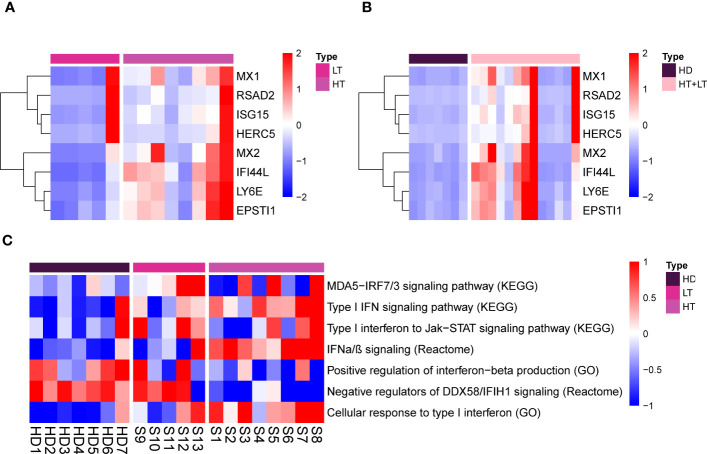
Type I IFN genes signature and representative pathways/biological functions in PBMCs of MDA5+ DM. Heatmap for eight type I-specific interferons response genes of HT vs. LT **(A)**, MDA5+ vs. HD **(B)**. Heatmap for particular biological functions and representative pathways related to type I IFN among three groups **(C)**. HD, healthy donors; HT, MDA5+ patients with high-titer of anti-MDA5 antibodies; LT, MDA5+ patients with low-titer of anti-MDA5 antibodies.

To further address type I IFN signature in detail, we performed interferons signature genes (ISG), defined by eight type I-specific interferons response genes. MDA+ DM patients showed elevated expression of these type I IFN signature genes, compared with healthy donors (**Figure 2**). Type I IFN marker genes showed differential regulation between patients in the high-titer and low-titer group (**Figure 2**). It is very interesting to note that one patient in the low antibody level group showed a very high type I IFN score. Lastly, we focus on these particular biological functions and extracted the most representative pathways that we visualized on a heatmap (**Figure 2C**).

### Clinical characteristics and prognosis of patients with MDA5+ DM

The clinical characteristics of 49 MDA5+ DM patients are shown in **Table 2**. The serum for detecting type I IFN score and anti-MDA5 antibody were simultaneously obtained and were performed on the same day or within 3 days of the laboratory testing without treatment modification. Pulmonary function tests and HRCT are also from the same period. Combined presence with anti-Ro52 antibodies was found in 61.2% of MDA5+ DM patients in our study. Almost all of these patients received combination therapy, including prednisolone, cyclophosphamide and calcineurin inhibitor (CNI). 88.8% of patients experienced intravenous immunoglobulin (IVIg) treatment and 37.8% used janus kinase inhibitor (JAKi). The average follow-up period was 17 (1, 56) (median (range)) months. Thirty-four patients survived the first 12 months without RP-ILD after disease onset (**Supplementary Figure S1**). No significant difference was observed with regard to the use of various drugs between the groups with RP-ILD and those without RP-ILD (**Supplementary Table S2**). The median anti-MDA5 titers at initial diagnosis tended to be higher, although not significantly (*P* = 0.172), in patients with occurrence of RP-ILD than those without RP-ILD in the first year (**Supplementary Figure S2**).

### Correlation of anti-MDA5 titer with the type I IFN score and clinical parameters in patients with MDA5+ DM

**Figure 3:**
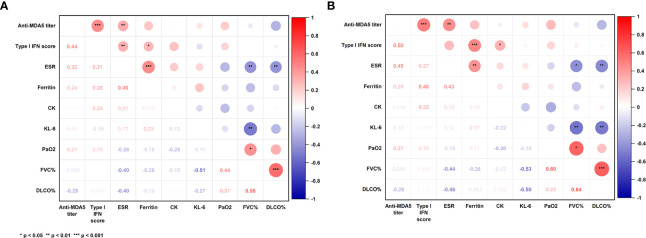
Correlation coefficients among concurrent clinical parameters in all specimens **(A)** and initial indicators of admission **(B)**. P-values were determined by Spearman’s correlation. The numerical labels are correlation coefficients. P< 0.05*, < 0.01**, < 0.001***. MDA5: melanoma differentiation-associated gene 5; IFN, interferon; ESR, erythrocyte sedimentation rate; CK: creatine kinase; KL-6, Krebs von den Lungen-6; PaO2, arterial oxygen pressure; FVC, forced vital capacity; Dlco, diffusing capacity of carbon monoxide.

**Figure 4:**
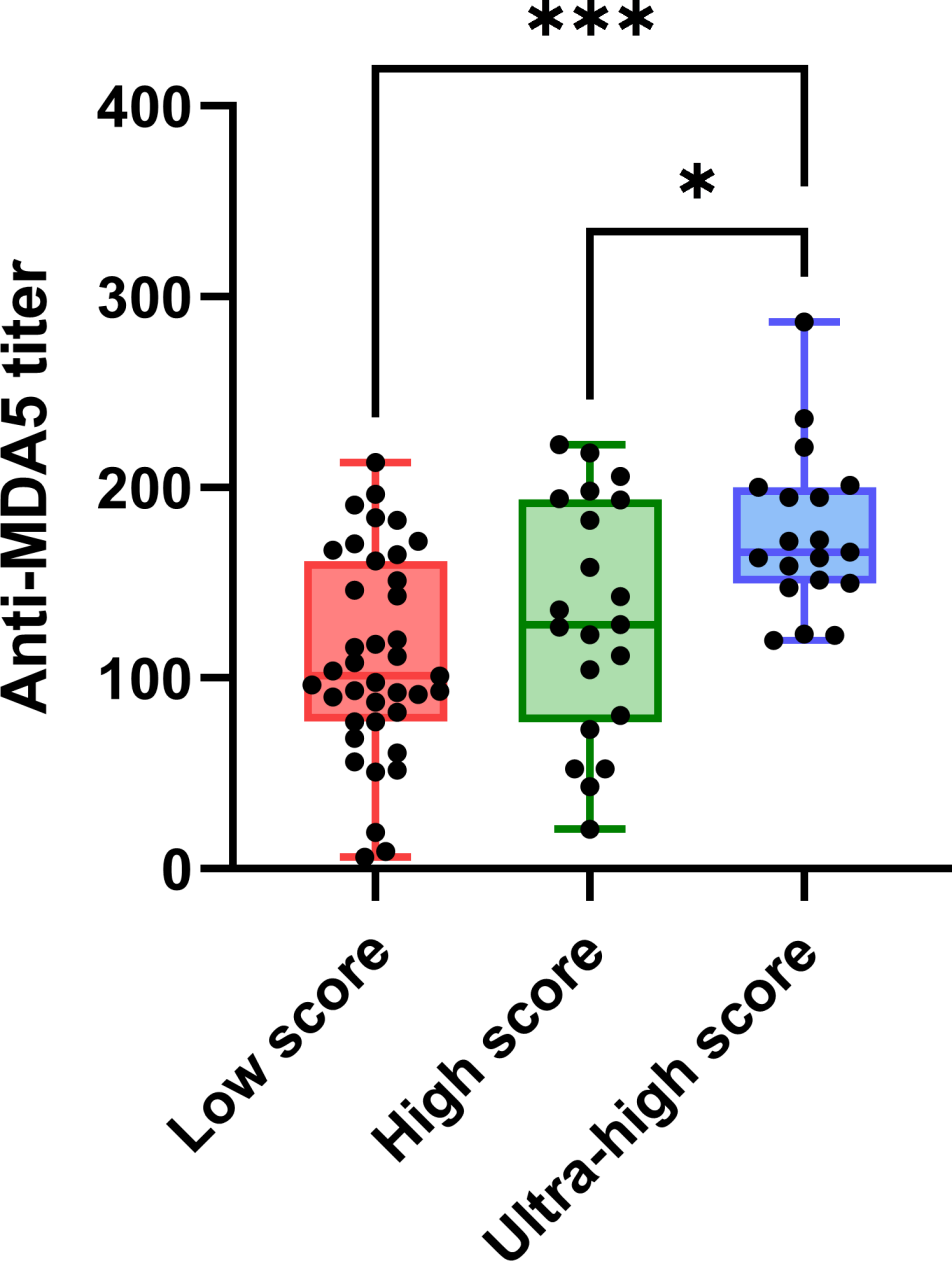
The comparation of anti-MDA5 titers among the groups of low (~P50), high (P50 to P75) and ultra-high (P75~). Anti-MDA5 titers were at the same time point as the type I interferon scores used for sub-group. Tukeys’ multiple comparisons test was used for multiple comparisons. P50: 5.52, P75: 18.13. P< 0.05*,< 0.001***. Horizontal bars: medians; bars: min to max; boxes: 25–75% range.

Correlation coefficients among concurrent clinical parameters were established in 79 specimens from 49 MDA5+ DM patients. Clinical parameters included anti-MDA5 titer, type I IFN score, ESR, ferritin, creatine kinase, Krebs von den Lungen-6 (KL-6), arterial oxygen pressure (PaO_2_) and pulmonary function parameters. Significant correlations were found between anti-MDA5 titers and type I IFN scores (r = 0.44, *P*< 0.001) in patients with MDA5+ DM (**Figure 3**). Furthermore, erythrocyte sedimentation rate (ESR) correlated with both anti-MDA5 titers (r = 0.32, *P* = 0.004) and type I IFN scores (r = 0.31, *P* = 0.005). Ferritin (r = 0.28, *P* = 0.024) was also found to correlate positively with type I IFN score (**Figure 3A**). Considering that the correlation between serum anti-MDA5 antibody levels and cell-based type I IFN scores may reflect the non-specific inhibitory effect of treatment, we further evaluated patients’ initial indicators of admission. A significant correlation between anti-MDA5 titer and type I IFN score remained (r = 0.05, P< 0.001). In addition, anti-MDA5 titer correlated with ESR (r = 0.45, P< 0.01). type I IFN score correlated with ferritin (r = 0.48, P< 0.001), creatine kinase (r = 0.32, P< 0.05) (**Figure 3B**). The anti-MDA5 titers were then compared among the three groups, which were divided into low (~P50), high (P50~P75), and ultra-high (P75~) according to the quartiles of type I IFN scores (**Supplementary Figure S3**). Contemporaneous anti-MDA5 titers were revealed to be significantly higher in the ultra-high group than the high (mean diff, 44.17; 95%CI, 4.10-84.24, *P* = 0.027) and low group (mean diff, 65.16; 95%CI, 29.76-100.60, *P*< 0.001) (**Figure 4**).

### Longitudinal dynamic evaluation of type I IFN scores and anti-MDA5 titers in patients with MDA5+ DM

**Figure 5:**
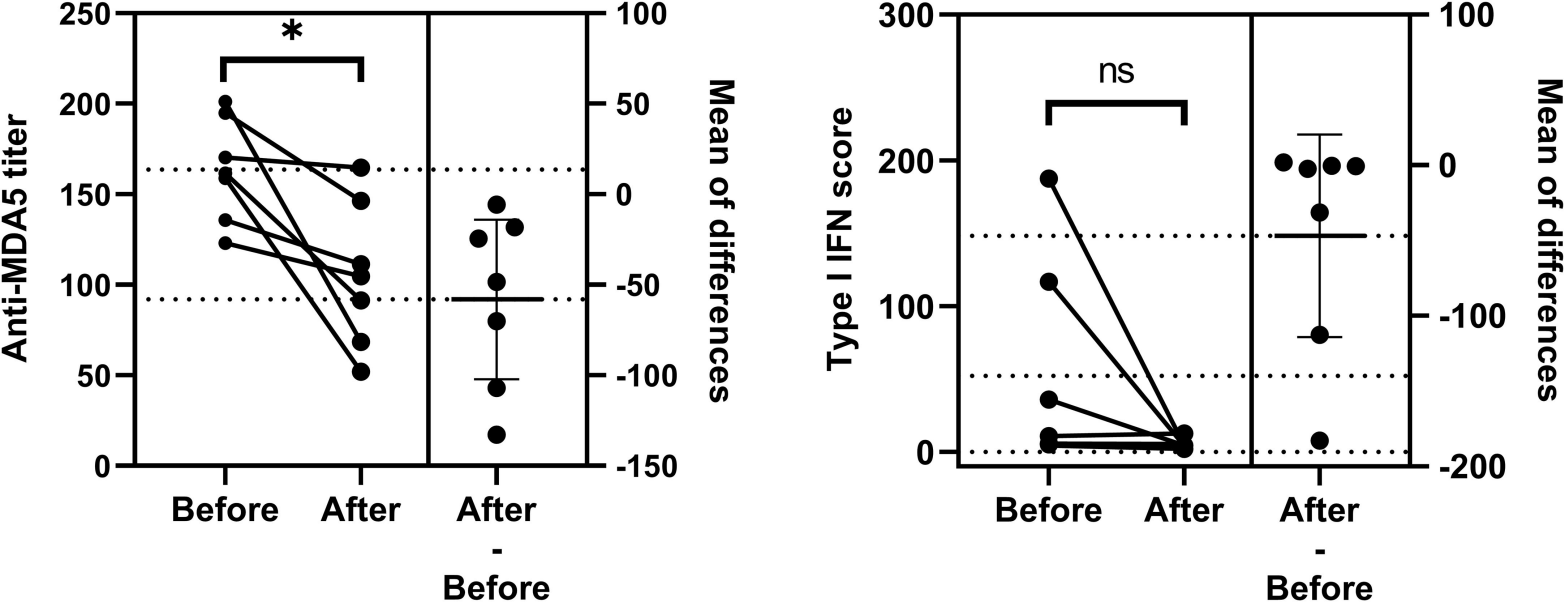
Comparation of anti-MDA5 titers and type I IFN scores between the two timepoints. Changes in two indicators before and after treatment for the first diagnosis are shown respectively. Comparisons were performed using Paired t-test. P< 0.05*. ns, no significance.

There were two to four dynamic measures available for analysis in a total of 14 patients. Seven of them were diagnosed as MDA5+ DM for the first time. We collected on-admission blood samples at the time of first diagnosis and second follow-up visit from these seven patients and assessed changes of anti-MDA5 titers and type I IFN scores between the two timepoints. Anti-MDA5 titers (mean diff, -58.12; 95%CI, -102.30 - -13.98, *P* = 0.018) showed differences before and after 39.14 ± 15.53 (mean ± SD) days of treatment, while type I IFN scores (mean diff, -46.93; 95%CI, -1114.20 - 20.31, *P* = 0.139) did not (**Figure 5**). Four patients, whose scores scarcely changed, had received hormones prior to their admission for initial diagnosis.

**Figure 6:**
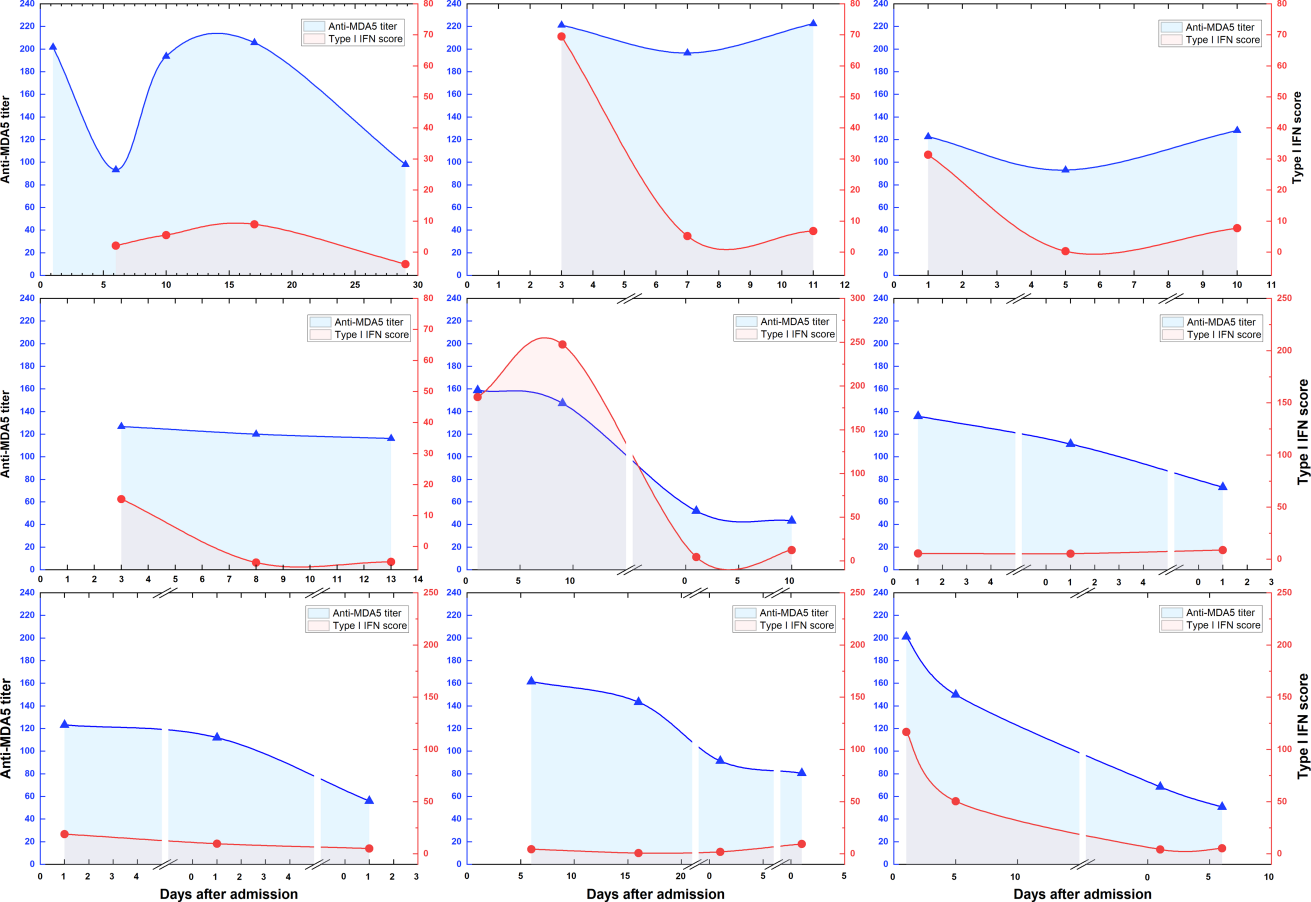
Dynamic changes of anti-MDA5 antibody titers and type I IFN scores during treatment. Overall dynamic changes of two indicators are shown according to the days of hospitalization for 9 patients with MDA5+ DM. The segments in the horizontal axis represents the next admission.

A total of 9 patients had 3 or more longitudinal specimens preserved during post-admission treatment. We traced the dynamic changes of anti-MDA5 antibody titers and type I IFN scores during treatment (**Figure 6**). These longitudinal evaluations suggest that anti-MDA5 antibody titers and type I IFN scores tend to normalize in most patients with MDA5+ DM after active treatment. In addition, the type I IFN scores showed an abrupt decrease at the beginning of treatment, and the anti-MDA5 titers presented a delayed and slower decreasing trend than the type I IFN scores.

## Discussion

MDA5+ DM tends to strike clinicians with a high incidence of severe morbidity and life-threatening RP-ILD, which occurred mainly in the early stages of the disease, especially within 6-12 months after diagnosis (23–25). In East Asia, 6-month mortality ranged from 33% to 66% even under intensive immunosuppressive therapy (26). Currently, more and more clinicians are focusing on MDA5, but the pathogenesis of the disease is still poorly understood, which leads to great challenges in clinical diagnosis and treatment.

Interferons (IFNs) are a superfamily of cytokines that mediate host antiviral responses and have pivotal roles in both innate and adaptive immunity responses. Three types of interferons, type-I, type-II, and type-III, bind to different heterodimeric receptors and induces transcription of hundreds of genes, termed as IFN stimulated genes (ISGs), through the Janus kinase-signal transducer and activator of transcription (JAK-STAT) signaling pathway (27, 28). Of these, type I IFNs, composed of IFNα, IFNβ and other IFNs, are the largest class of IFNs (5). In recent years, the role of the type I IFN signaling pathway in the pathogenesis of MDA5+ DM has extensively aroused interest. Here, we report viruses and cytokines-related genes and pathways were markedly upregulated in patients with MDA5+ DM according to our RNA sequencing data, which is similar to the previous transcriptome findings (29). A recent study for over-stimulated type I IFN signaling, in peripheral B and T cells and lung cells, have also revealed a critical role of type I IFN for MDA5+ DM via single-cell RNA sequencing (30). It also showed that an exacerbated humoral response existed and fibroblasts had strong type I IFN signaling interactions with antibody-secreting cell, which raises an interesting possibility for our conceptualization of the linkage between autoantibodies and type I IFN.

Anti-MDA5 antibody, which is the centerpiece of the MDA5+ DM, has been found to be present positive but low-grade in some other diseases, such as systemic lupus erythematosus (SLE) and COVID-19 (17, 31). It is interesting to note that they found a correlation between anti-MDA5 antibodies and type I IFN signaling in SLE patients, but no correlation was observed in DM. Additional studies have also observed that no significant association between antibodies and other clinical parameters (31). However, compared to their study, our study has included ESR, a key indicator of inflammation, and has a larger clinical sample including longitudinal samples, which may account for the correlation conclusion in our study. We conjecture that the possible reason for this discrepancy is that the severity and course of the disease varies considerably between individual patients, but the dynamics of the indicators may be linked and not exactly synchronized.

Several studies concluded that high-titer is an independent risk factor for poor prognosis (32–34), even associated with IgG subtypes (35, 36), but a different opinion has been noted in other studies (37). In our study, there was no significant difference of antibody titers at the first diagnosis between subgroups with regard to prognosis, although it appeared to differ. We chose the time-point of initial diagnosis to assess antibody titers, due to the dynamics of antibody titers according to our study and previous studies. As shown in a recent study on MDA5 subgroup analysis, a higher proportion of patients with high-titer anti-MDA5 was found in the subgroup with high mortality, and myasthenia, rash, arthritis and anti-Ro52 were also most prevalent in the subgroup with high mortality (24). This seems to be in line with the clinical differences reported in our study.

Previous studies have found that autoantibodies in MDA5+ DM patients induce IFN-γ secretion by peripheral monocytes (38), and immune complex formed by MDA5 and anti-MDA5 antibody can stimulate IFN-α production by plasmacytoid dendritic cells (31). Together with the molecular links of MDA5/MAVS and IFN/JAK/STAT pathway, which all pointed to an interesting link between autoantibody responses and type I IFN signaling. According to transcriptome, we highlighted the upregulation of a type I interferons-specific signature, which was seen not only in patients with MDA5+ DM but also in the high-titer group. Additionally, a significant correlation between anti-MDA5 and type I IFN was observed in our dynamic data. Given the inclusion of longitudinal data during treatment, a proportion of patients had low-score for type I IFN. For this reason, we further divided them into low, high and ultra-high scores based on quartiles and found that patients with ultra-high scores had higher levels of antibodies. In addition, it is worth noting that the correlation between ferritin and type I IFN. This seems to present consistency with the opinions on hyperferritinemia that ferritin and pro-inflammatory cytokines may induce a vicious cycle that ultimately leads to the development of cytokine storm syndrome in some systemic autoimmune disorders (39). Besides, taken together with the above-mentioned elevation of anti-MDA5 antibodies in COVID-19, the finding that hyperferritinemia paralleled with high levels of inflammatory mediators in the peripheral blood in patients with COVID-19 seems to reinforce the similarity between the two diseases and points to the possible involvement of viral infection in the pathogenesis of MDA5+ DM.

Our study is consistent with previous evidence that anti-MDA5 titers gradually decrease in response to treatment (32, 37), while we also found that IFN levels generally dropped to a state of low activity within a few days after application of steroids and immunosuppressants. The lag in antibody response over IFN signaling in our research may correspond to the increase of cytokine levels after positive anti-MDA5 antibody titers indicated by other studies (33). To note, both indicators may behave with disparate response at the time of relapse or persistent over-activation as they did at remission, which is a conjecture based on the case of patient S13 in our transcriptomic data, with a concurrent low antibody titer status and high type I IFN signaling. Aberrant increase of antibody titers may signal disease relapse (32). While type I IFN has been extensively established as a hallmark of MDA5+ DM, it remains to be further investigated whether the inconsistency between strong interferon signaling and low-titer antibodies mirrors disease flare.

JAK-STAT is one of the key pathways for responding to and transducing inflammatory signals from extracellular ligands such as IFN. Combined tofacitinib therapy has also been found to be effective in improving the prognosis of patients with MDA5+DM (40), even for refractory patients (41). Tofacitinib inhibits JAK kinases and block downstream pathways by multiple cytokines, such as types I IFNs. There was no difference in the rate of jak inhibitor administration between our two groups in terms of prognosis. In conclusion, there are no data for reference from randomized controlled studies or large-scale observational studies. So, our dynamic analysis, presenting a prompt decline after treatment and maintaining prolonged low levels, may provide inspiration for future studies that early treatment with JAK inhibitors may be more beneficial.

There are several limitations in the current study. First, due to limitations in the specimen size, we failed to assess the dynamics of transcriptomics over the treatment process on the same patients and use dynamic changes of anti-MDA5 antibody titers to assess treatment response and predict clinical course. Second, the relationship between autoantibody and type I IFN signaling was only assessed by serum and PBMC, without further validation of the association by *in vitro* cellular assays. Third, the current experiments only provide evidence for speculating anti-MDA5 autoantibodies and type I IFN as the central mechanism of this disease, but do not address the role of JAK/STAT and MDA5/MAVS pathways in this disease. These limitations will be addressed in our future studies by establishing long-term cohorts with more patients and *in vitro* experiments.

In summary, type I IFN signature are likely to be a hallmark of MDA5+ DM, and type I IFN feature molecules seemed weaker in the patients with low-titer antibodies, which reveal possibly modification along with tailored treatment. Real-time monitoring of anti-MDA5 titers and type I IFN signature appeared to be a pattern of similar trends, yet possibly the response of interferon was more rapid. It is necessary to dissect the linking of tendency between type I IFN response and anti-MDA5 antibodies, as these may provide a context for the investigation into the underlying mechanisms of MDA5+ DM, a serologically-defined disease, and offer promising targets and a well-placed therapeutic window.

## Data availability statement

The data presented in the study are deposited in the Sequence Read Archive (SRA) repository, accession number PRJNA1001406.

## Ethics statement

The studies involving humans were approved by Ethics Committee of the First Affiliated Hospital of Zhengzhou University. The studies were conducted in accordance with the local legislation and institutional requirements. The participants provided their written informed consent to participate in this study. Ethical approval was not required for the studies on animals in accordance with the local legislation and institutional requirements because only commercially available established cell lines were used.

## Author contributions

Conceptualization and design: YW and GZ. Performing of experiments: YW, HL, MT. Acquisition of clinical data: YW, HJ, JL, JC. Analysis and manuscript drafting: YW. Critical revision of the manuscript for content: HJ, MT, JL, JC, GZ. All authors contributed to the article and approved the submitted version.

## Supplementary material

Supplementary Table S1 Type I Interferon inducible genes primer sequences. $IFN\;score\;=\;\sum \frac{Gene\;MDA5+DM\;-\bar{x}\left(GeneHD\right)}{SD\left(GeneHD\right)}$ Gene represents the relative expression of a specific gene in MDA5+DM patients or healthy donors (n = 20). The type I IFN score was calculated as the sum of the three normalized expression levels.

Supplementary Table 2 Antifibrotic drugs: included pirfenidone or nintedanib; Others refer to methotrexate, mycophenolate mofetil, hydroxychloroquine or tocilizumab; GCs dosage: converted to prednisone dosage. RP-ILD, rapidly progressive interstitial lung disease; BMI, body mass index; ANA, antinuclear antibodies; anti-MDA5, anti-melanoma differentiation-associated protein-5; GCs, glucocorticoids; IVIg, intravenous immunoglobulins; CYC, Cyclophosphamide; JAKi, Janus kinase inhibitors.

Supplementary Figure 1 Cumulative rate of RP-ILD for patients with MDA5+DM. The cumulative 60-month rates were calculated using the Kaplan-Meier test. RP-ILD, rapidly progressive interstitial lung disease.

Supplementary Figure 2 Comparation of Anti-MDA5 titers at initial diagnosis between patients with RP-ILD and Non-RP-ILD. Comparation was performed using t-test. ns: no significance. RP-ILD, rapidly progressive interstitial lung disease.

Supplementary Figure 3 Distribution of type I IFN scores in the interquartile ranges. Median: 5.52, P75: 18.13.
