# Supplementary material for: Transcriptomic profiling and longitudinal study reveal the relationship of anti-MDA5 titer and type I IFN signature in MDA5+ dermatomyositis
Source: Front Immunol. 2023 Aug 28;14:1249844. doi: 10.3389/fimmu.2023.1249844 (PMC10494241; doi:10.3389/fimmu.2023.1249844)
Supplement: Supplementary Table S1 — Type I Interferon inducible genes primer sequences. IFN score = ∑Gene MDA5+DM −x¯(GeneHD)SD(GeneHD) Gene represents the relative expression of a specific gene in MDA5+DM patients or healthy donors (n = 20). The type I IFN score was calculated as the sum of the three normalized expression levels. [file DataSheet_1.doc]

Supplementary data

Supplementary Table S1 Type I interferon inducible genes primer sequences

| Primer | Forward Sequence (5'-3') | Reverse Sequence (5'-3') |
| --- | --- | --- |
| GAPDH | CAACGGATTTGGTCGTATT | GATGGCAACAATATCCACTT |
| MX1 | TACCAGGACTACGAGATTG | TGCCAGGAAGGTCTATTAG |
| PKR | CTTCCATCTGACTCAGGTTT | TGCTTCTGACGGTATGTATTA |
| IFIT1 | CTCCTTGGGTTCGTCTATAAATTG | AGTCAGCAGCCAGTCTCAG |

IFN score = ∑
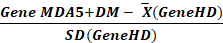


Gene represents the relative expression of a specific gene in MDA5+DM patients or healthy donors (n = 20). The type I IFN score was calculated as the sum of the three normalized expression levels.

Supplementary Table S2

|  | RP-ILD (N = 15) | Non-RP-ILD (N = 34) |
| --- | --- | --- |
| Age, mean ± SD, years | 51.60 ± 11.50 | 50.38 ± 11.03 |
| Sex (female/male) | 8/7 | 26/8 |
| Former or current smoker, n (%) | 2 (13.3%) | 1 (2.9%) |
| BMI, median (IQR), kg/m^2^ | 24.65 (22.30, 26.27) | 23.63 (21.49, 26.90) |
| Myasthenia, n (%) | 11 (73.3%) | 24 (70.6%) |
| Rash, n (%) | 11 (73.3%) | 30 (88.2%) |
| cough or dyspnea on exertion, n (%) | 14 (93.3%) | 25 (73.5%) |
| Arthritis, n (%) | 7 (46.7%) | 22 (64.7%) |
| ANA>1:100, n (%) | 2 (13.3%) | 5 (14.7%) |
| Anti-Ro52 positive, n (%) | 13 (86.7%) | 17 (50.0%) |
| Anti-MDA5 [titer](../../../../D:/app/baidu-translate-client/resources/app.asar/app.html) at initial diagnosis, median (IQR), U/ml | 171.85 (158.28, 201.19) | 169.00 (134.75, 194.12) |
| Treatment |  |  |
| Initial GCs dosage, median (range), mg/day | 100.00(75.00, 200.00) | 100 (50.00, 200.00) |
| IVIg, n (%) | 100 (100.0%) | 28 (82.4%) |
| Tacrolimus, n (%) | 11 (73.3%) | 22(64.7%) |
| Cyclosporine A, n (%) | 0 | 7 (20.6%) |
| CYC, n (%) | 10 (66.7%) | 15 (44.1%) |
| JAKi, n (%) | 7 (46.7%) | 12 (35.3%) |
| Antifibrotic drugs, n (%) | 8 (53.3%) | 11 (32.4%) |

Antifibrotic drugs: included pirfenidone or nintedanib; Others refer to methotrexate, mycophenolate mofetil, hydroxychloroquine or tocilizumab; GCs dosage: converted to prednisone dosage.

RP-ILD, rapidly progressive interstitial lung disease; BMI, body mass index; ANA, antinuclear antibodies; anti-MDA5, anti-melanoma differentiation-associated protein-5; GCs, glucocorticoids; IVIg, intravenous immunoglobulins; CYC, Cyclophosphamide; JAKi, Janus kinase inhibitors.


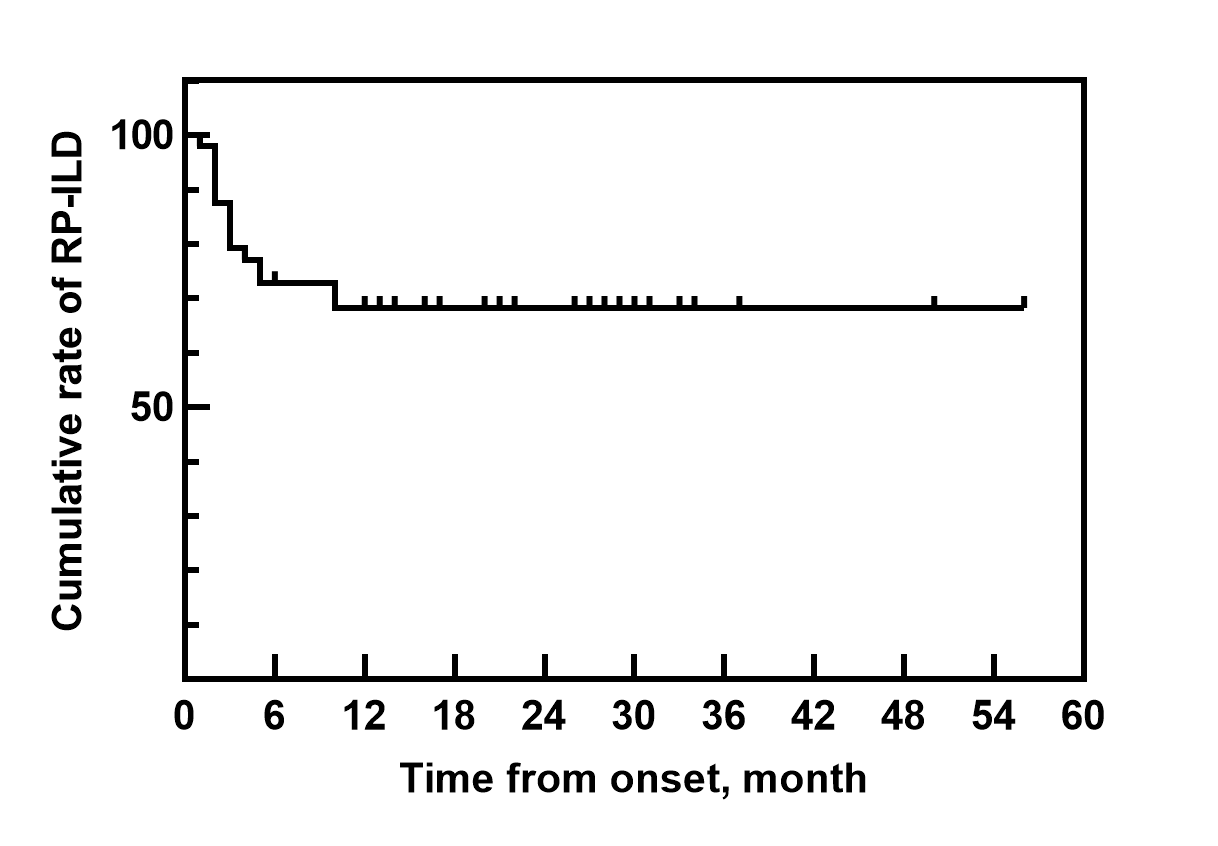


**Supplementary Figure S**1 **Cumulative rate of RP-ILD for patients with MDA5+DM.** The cumulative 60-month rates were calculated using the Kaplan-Meier test. RP-ILD, rapidly progressive interstitial lung disease


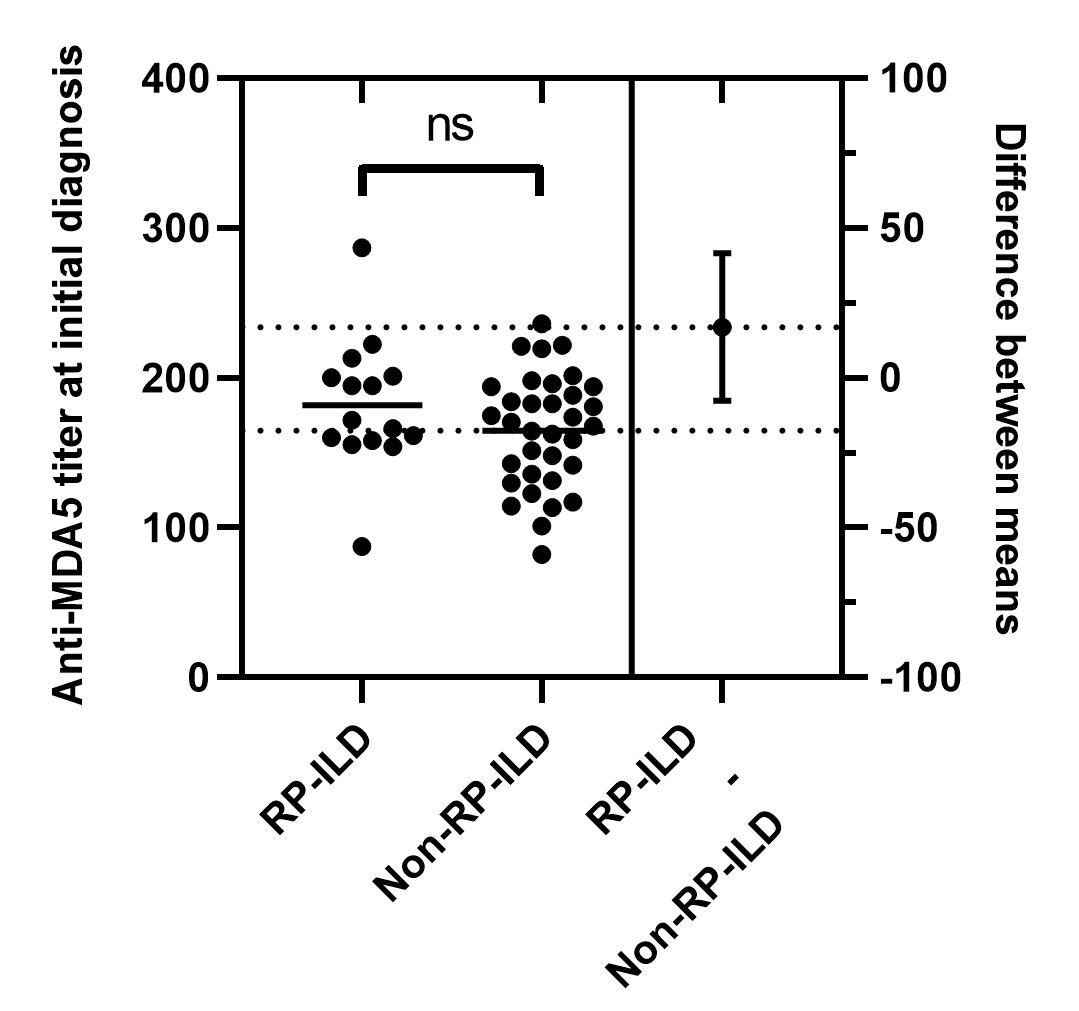


**Supplementary Figure S2** **Comparation of Anti-MDA5 titers at initial diagnosis between patients with RP-ILD and Non-RP-ILD.** Comparation was performed using t-test. ns, no significance. RP-ILD, rapidly progressive interstitial lung disease


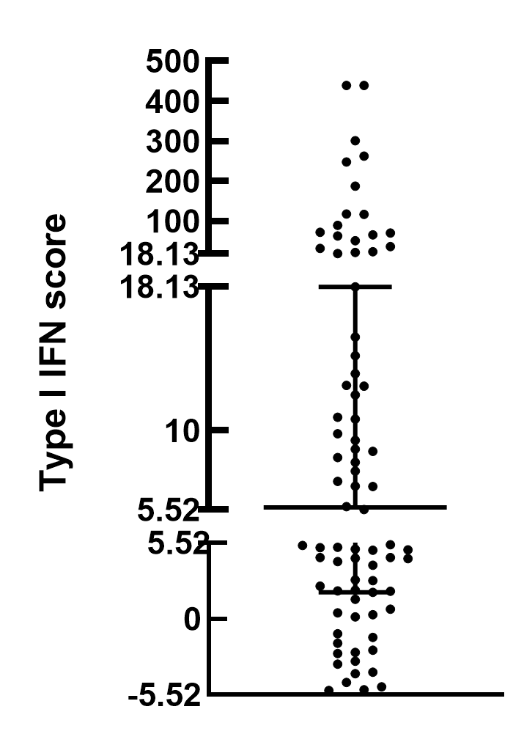


**Supplementary Figure S3 Distribution of type I IFN scores in the interquartile ranges.** Median: 5.52, P75: 18.13.
